# Novel High-Quality Amoeba Genomes Reveal Widespread Codon Usage Mismatch Between Giant Viruses and Their Hosts

**DOI:** 10.1093/gbe/evae271

**Published:** 2025-01-06

**Authors:** Anouk Willemsen, Alejandro Manzano-Marín, Matthias Horn

**Affiliations:** Centre for Microbiology and Environmental Systems Science, Division of Microbial Ecology, University of Vienna, Vienna 1030, Austria; Centre for Microbiology and Environmental Systems Science, Division of Microbial Ecology, University of Vienna, Vienna 1030, Austria; Centre for Microbiology and Environmental Systems Science, Division of Microbial Ecology, University of Vienna, Vienna 1030, Austria

**Keywords:** amoeba genomes, giant viruses, codon usage, virus–host interactions

## Abstract

The need for high-quality protist genomes has prevented in-depth computational and experimental studies of giant virus–host interactions. In addition, our current knowledge of host range is highly biased due to the few hosts used to isolate novel giant viruses. This study presents 6 high-quality amoeba genomes from known and potential giant virus hosts belonging to 2 distinct eukaryotic clades: *Amoebozoa* and *Discoba*. We employ their genomic data to investigate the predictability of giant virus host range. Using a combination of long- and short-read sequencing, we obtained highly contiguous and complete genomes of *Acanthamoeba castellanii, Acanthamoeba griffini*, *Acanthamoeba terricola*, *Naegleria clarki*, *Vermamoeba vermiformis*, and *Willaertia magna*, contributing to the collection of sequences for the eukaryotic tree of life. We found that the 6 amoebae have distinct codon usage patterns and that, contrary to other virus groups, giant viruses often have different and even opposite codon usage with their known hosts. Conversely, giant viruses with matching codon usage are frequently not known to infect or replicate in these hosts. Interestingly, analyses of integrated viral sequences in the amoeba host genomes reveal potential novel virus–host associations. Matching of codon usage preferences is often used to predict virus–host pairs. However, with the broad-scale analyses performed in this study, we demonstrate that codon usage alone appears to be a poor predictor of host range for giant viruses infecting amoeba. We discuss the potential strategies that giant viruses employ to ensure high viral fitness in nonmatching hosts. Moreover, this study emphasizes the need for more high-quality protist genomes. Finally, the amoeba genomes presented in this study set the stage for future experimental studies to better understand how giant viruses interact with different host species.

**Significance**

Despite their environmental and evolutionary significance, high-quality genomes of unicellular eukaryotes such as amoebae are scarce. Amoebae can be associated with various types of symbionts, such as bacteria, fungi and viruses. They constitute a favorable environment for genetic exchange between sympatric symbionts, resulting in organisms with complex chimeric genomes, such as giant viruses. While few cultured amoeba strains serve as hosts for these viruses, their natural host range remains unclear. Similarities in codon usage are often used to predict viable virus–host pairs. However, with the high-quality amoeba genomes generated in this study, we demonstrate that codon usage alone cannot fully explain the known giant virus–host associations, likely resulting from their genome complexity. Our work provides valuable genomic data for amoebae, deepens our understanding of how to reveal novel virus–host pairs, and sets the stage for experimental studies to further investigate the interaction of amoebae, their viruses and other symbionts.

## Introduction

Protists are a diverse group of microorganisms representing various evolutionary lineages in the eukaryotic tree of life (Burki 2014; Simpson et al. 2017). They are also defined as eukaryotes that are not animals, plants, or fungi, with most being single-celled organisms. Amoebae are polyphyletic (Burki et al. 2020) and are among the protist members that can migrate by a process known as amoeboid movement (Webb and Horwitz 2003; Yoshida and Soldati 2006). They are present in various environments, from soil and freshwater to marine habitats, and their extensive genetic diversity reflects their diverse ecological niches and lifestyles. Some amoebae are known to cause disease in humans. For example, certain members of the family *Acanthamoeba* are causal agents of a severe sight-threatening infection of the cornea (Lorenzo-Morales et al. 2015), and the so-called “brain-eating” amoeba *Naegleria fowleri* can cause a rare but nearly always fatal brain infection (Grace et al. 2015). Amoebae can be associated with various types of symbionts, such as bacteria (Horn and Wagner 2004; Molmeret et al. 2005; Shi et al. 2021), algae (Weiner et al. 2022), viruses (Oliveira et al. 2019), and fungi (Steenbergen et al. 2001; Corsaro et al. 2014). Interaction with these symbionts can be mutualistic, parasitic, or commensal. With amoebae hosting such a variety of organisms, they constitute a favorable environment for genetic exchange between sympatric symbionts resulting in organisms with complex chimeric genomes (Moreira and Brochier-Armanet 2008; Boyer et al. 2009; Moliner et al. 2010; Wang and Wu, 2017), such as giant viruses.

Giant viruses are a highly unusual group of viruses, of which the first member was discovered only 2 decades ago (La Scola et al. 2003; Raoult et al. 2004). These viruses are classified within the phylum *Nucleocytoviricota*, an assemblage of several families of double-stranded DNA (dsDNA) viruses infecting multicellular and unicellular eukaryotes. As their name suggests, giant viruses distinguish themselves from other viruses with vast particle and genome sizes (La Scola et al. 2003; Philippe et al. 2013; Legendre et al. 2014; Abrahão et al. 2018). The size of their virions exceeds that of the smallest known bacteria and archaea. Their large genomes revealed an unexpected complexity, including the existence of hundreds of genes that have not yet been attributed to viruses (Schulz et al. 2017; Abrahão et al. 2018; Needham et al. 2019a; Brahim Belhaouari et al. 2022). Interestingly, giant viruses do not form a monophyletic clade within the *Nucleocytoviricota* and appear to have evolved on multiple independent occasions from smaller viruses (Koonin and Yutin 2018). While the evolutionary factors that promote genome expansion in giant viruses remain unclear, it is suggested that virus–host interactions may play an important role where viral size limitations imposed by multicellular hosts appear to restrict giant viruses to unicellular eukaryotic (i.e. protist) hosts (Koonin et al. 2020). Yet, the natural host species and precise host range remain unknown for most giant viruses.

Our current knowledge of giant virus host range is strongly biased toward lytic viruses that have been isolated through co-cultivation with a limited number of protists, mainly with *Acanthamoeba* species (La Scola et al. 2003; Boyer et al. 2009; Legendre et al. 2014, 2015; Abrahão et al. 2018; Yoshikawa et al. 2019) but also with members of the genera *Vermamoeba* (Reteno et al. 2015; Bajrai et al. 2016; Abrahão et al. 2018), *Bodo* (Deeg et al. 2018), *Cafeteria* (Fischer et al. 2010), and more recently *Naegleria* (Arthofer et al. 2024). Yet, metagenomic studies suggest that *Acanthamoeba*-associated giant viruses are less prevalent under natural conditions. Rather, these studies suggest other amoeboid flagellates and algae species as natural hosts (Zhang et al. 2015; Schulz et al. 2017; Needham et al. 2019b).

In several organisms, it has been shown that a correlation between codon usage and tRNA content exists (Anderson 1969), where coevolution of codon usage and tRNA content can optimize the efficiency of translation (Ikemura 1985; Rocha 2004; Higgs and Ran 2008). Most known viruses have highly compact genomes that do not encode tRNAs. Thus, the translation of viral proteins relies on the host tRNA pool. This situation creates translational selection for the adaptation of viral codon usage to those of their hosts, ensuring efficient viral translation. Therefore, the prediction of viable virus–host pairs is often based on similarities in codon usage. This method works well for phages, as there is generally a strong similarity in codon usage between prokaryotes and these viruses (Sau et al. 2005; Lucks et al. 2008; Esposito et al. 2016). However, for most eukaryotes (animals and plants), there appears to be an overall poor match of codon usage with their infecting viruses (Simón et al. 2021). Among protists, it was even noted that there is a negative correlation (Simón et al. 2021). For certain giant viruses, the presence of tRNAs and other translation-related genes in their genomes (Raoult et al. 2004; Schulz et al. 2017; Abrahão et al. 2018; Koonin and Yutin 2018) might explain how these viruses can thrive well in protist hosts. Indeed, for one large DNA virus (*Ostreococcus tauri virus 5*) infecting a marine green alga (*O. tauri*), it has been described that viral tRNAs complement the host tRNA pool for translational optimization of the viral genes (Michely et al. 2013). Unfortunately, the lack of data does not allow for an accurate portrait of virus-protist interactions at the genomic level.

While high-quality genomic resources for diverse giant virus lineages are currently available, a significant gap exists regarding similar resources for their hosts. Thus, in this study, we sequence and provide high-quality genomes of 6 amoeba that we currently use in our laboratory to study their interactions with giant viruses: *Acanthamoeba terricola* Neff (up to recently classified as *Acanthamoeba castellanii* Neff (Corsaro et al. 2024)), *A. castellanii* 1BU, *Acanthamoeba griffini* Sawyer, *Vermamoeba vermiformis* CDC-19, *Naegleria clarki* RU30, and *Willaertia magna* T5(S)44. These amoebae belong to 2 distantly related eukaryotic clades: the *Amoebozoa* within the Amorphea supergroup and *Discoba* within the unresolved Excavates supergroup (Burki et al. 2020). Yet, despite their unrelatedness, most of these amoebae can host giant viruses, reflecting the remarkable structural and genomic diversity we observe among giant viruses (Koonin and Yutin 2018; Aylward et al. 2021; Fischer et al. 2023). To investigate whether codon usage plays a vital role in giant virus host adaptation, we compared the codon usage preferences of both partners. In addition, we also examine the amoeba genomes for viral integrations, as some of these have been shown to act as antiviral defense systems (Fischer and Hackl 2016; Levasseur et al. 2016) and have the potential to reveal novel giant virus–host associations (Maumus and Blanc 2016; Bellas et al. 2023). Finally, we evaluate additional important factors for predicting giant virus host range and discuss potential strategies that giant viruses employ to optimize viral fitness in their hosts.

## Results and Discussion

### The 6 Amoeba Genomes are Highly Contiguous and Complete

We used a combination of Illumina short-reads and Oxford Nanopore long reads to assemble the amoeba genomes (see Materials and Methods). For all 6 amoebae, the genome assemblies were contained in a low number of scaffolds (Table 1), with 90% of the individual genomes contained within 57 (*A. terricola* Neff), 123 (*A. castellanii* 1BU), 280 (*A. griffini* Sawyer), 132 (*V. vermiformi*s CDC-19), 559 (*W. magna* T5(S)44), and 670 (*N. clarki* RU30) scaffolds. These are the first published genome assemblies of *A. castellanii* 1BU, *A. griffini*, *N. clarki*, and *W. magna* T5(S)44. For *V. vermiformis* and *W. magna,* the assemblies significantly improve the number of contigs and contiguity compared to previously published assemblies of the same species (supplementary table S1, Supplementary Material online). Compared to the *A. terricola* Neff reference assembly, this assembly is slightly longer with fewer contigs (supplementary table S1, Supplementary Material online). Recently, a somewhat longer (43.8 Mb) *A. terricola Neff* assembly was published, where with the inclusion of Hi-C, the assembly was less fragmented and resulted in 111 scaffolds (Matthey-Doret et al. 2022).

**Table 1:** Genome assembly statistics for the final assemblies of the 6 protist genomes

Species	Size (Mbp)	Scaffolds (No.)	N50 (Mbp)	L50 (No.)	L90 (No.)	Largest sequence (Mbp)	Total GC%	protein-coding genes (No.)
*A. terricola* Neff	42.97	246	0.9	17	57	1.9	58.42	14,924
*A. castellanii* 1BU	47.37	474	0.5	25	123	2.0	58.53	14,761
*A. griffini* Sawyer	52.91	862	0.4	33	280	1.5	56.66	16,497
*V. vermiformis* CDC-19	43.35	309	0.4	39	132	0.8	41.94	15,385
*N. clarki* RU30	53.71	1,709	0.1	109	670	1.2	32.27	22,696
*W. magna* T5(S)44	47.61	1,239	0.09	140	559	0.6	24.69	18,644

**Fig. 1.**
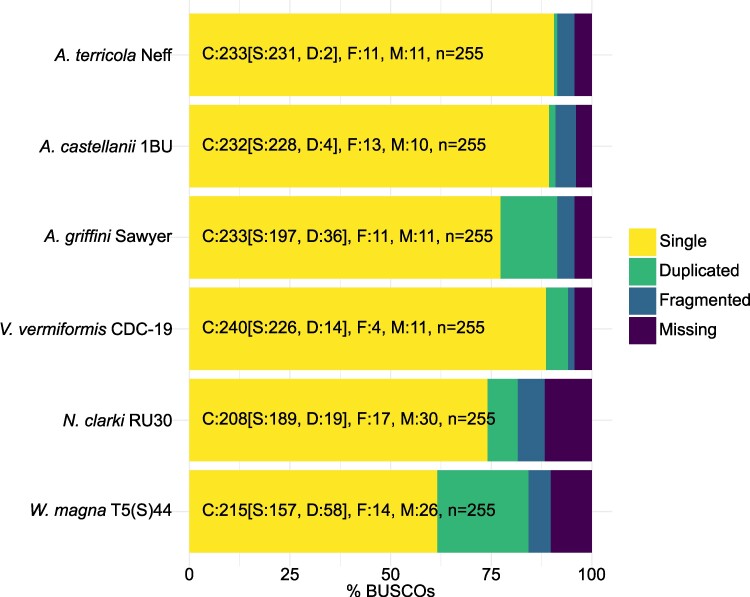
Genome completeness (BUSCO scores) for the annotated genomes of the 6 amoebae. BUSCO was run using the transcriptome mode against the eukaryotic database (C: complete [D: duplicated], F: fragmented, M: missing, *n*: total BUSCO groups searched). These results indicate high completeness levels of between 81.6% and 94.1%.

Genome completeness was estimated based on single-copy orthologs using BUSCO scores (Simão et al. 2015). These were calculated for the genome assemblies and the genome annotations at the protein and transcript levels. The completeness score for the genomes varied between 78.1% and 86.6% of complete eukaryotic universal single-copy orthologs (supplementary table S2, Supplementary Material online). For most genomes, these scores improved notably when considering the annotated proteins (supplementary table S2, Supplementary Material online) and transcripts (Fig. 1 and supplementary table S2, Supplementary Material online), with for the transcripts scores of 94.1% (*V. vermiformis*), 91.4% (*A. terricola* Neff, *A. griffini*), 91.0% (*A. castellanii* 1BU), 84.3% (*W. magna*), and 81.6% (*N. clarki)*. Only the annotated *W. magna* and *N. clarki* genomes appear to be missing some BUSCO gene matches that are, in fact, present in the genome (supplementary table S2, Supplementary Material online), as their genome assembly completeness scores are higher (86.6% and 85.1%, respectively). This incongruency reveals the limitations of the gene prediction tool we used for amoebae in the *Discoba* clade.

### Phylogenetic and Phylogenomic Analyses Define Amoeba Relationships

From each genome sequenced in this study, 18S rRNA sequences were extracted and aligned with other complete and near-complete protist 18S rRNA sequences. Phylogenetic trees were constructed for the phyla *Discosea* (supplementary fig. S1, Supplementary Material online; including *Acanthamoeba* spp.), *Tubulinea* (supplementary fig. S2, Supplementary Material online; including *V. vermiformis*), and *Heterolobosea* (supplementary fig. S3, Supplementary Material online; including *N. clarki* and *W. magna*). These trees demonstrate that on the 18S rRNA level, the amoebae cluster within their expected clades with their close relatives. Nonetheless, 18S rRNA gene sequences are insufficient to resolve amoeba at the species level as the identity between closely related strains is high (supplementary Data, Supplementary Material online at Zenodo repository). Despite the high 18S rRNA sequence identity, a previous study has already shown considerable variation in the mitochondrial protein-coding genes and was thus able to uncover diversity even between conspecific *Acanthamoeba* and *Vermamoeba* (Fučíková and Lahr 2016). We identified a unique region in the mitochondrial genome of *W. magna* T5(S)44 sequenced in this study, containing 2 hypothetical proteins shared with *Naegleria* spp., allowing us to distinguish this strain from other *W. magna* strains (supplementary Data, Supplementary Material online at Zenodo repository). These results stress the need for more high-quality complete genome sequences to perform genome-scale analyses and resolve relationships among amoebae.

**Fig. 2.**
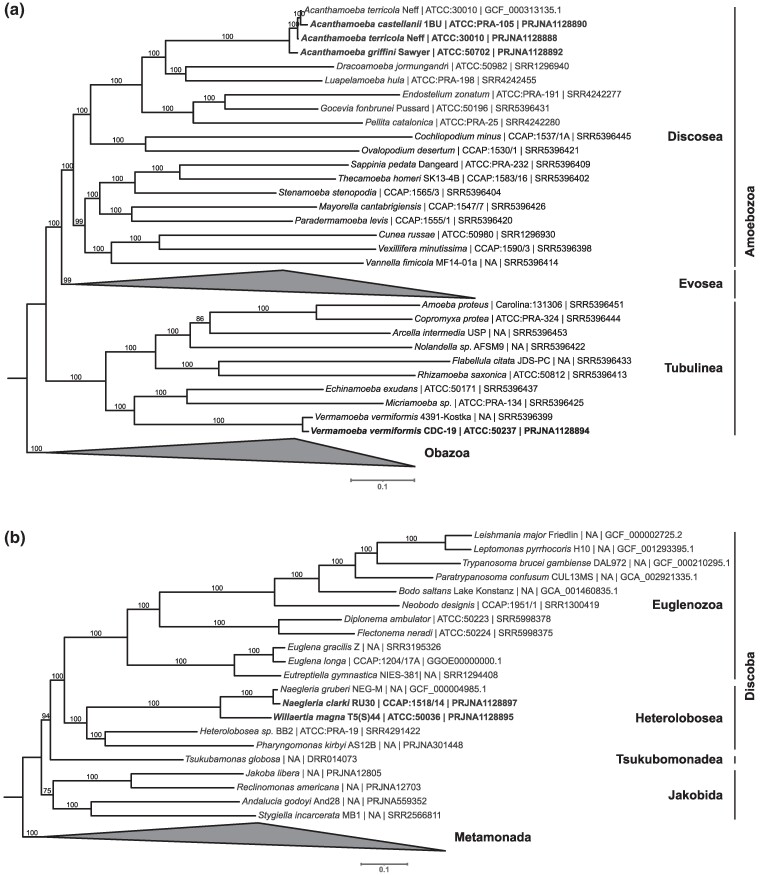
Phylogenomic trees inferred for 2 eukaryotic clades that include the newly sequenced amoebae. a) This maximum likelihood phylogenomic tree for *Amoebozoa* was inferred from 228 proteins and 52 taxa, using the LG+R7 model of evolution. The names of the clades representing different phyla within the *Amoebozoa* are indicated on the right side. Members representing different phyla within the *Obazoa* clade (6 taxa) were used as an outgroup. b) This maximum likelihood phylogenomic tree for *Discoba* was inferred from 228 proteins and 24 taxa, using the LG+F+R5 model of evolution. The names of the clades representing different phyla within the *Discoba* are indicated on the right side. Members representing different phyla within the *Metamonada* clade (3 taxa) were used as an outgroup. For both trees, bootstrap support values are given on the branches, and the names on the leaves are composed of “species names [strain] | culture collection database:accession number | NCBI dataset accession number” (see supplementary table S3, Supplementary Material online). The newly added taxa are indicated in bold font.

To contribute to the collection of sequences for the eukaryotic tree of life, we used the package PhyloFisher (Tice et al. 2021) and its associated database for phylogenomic analyses. The resulting phylogenomic trees of the *Amoebozoa* (Fig. 2a) and *Discoba* (Fig. 2b) eukaryotic clades show high levels of bootstrap support, where the *Acanthamoeba*, *Vermamoeba*, and *Naegleria* sequences form separate monophyletic clades, and *Willaertia* appears as a sister taxon to the 2 available *Naegleria* species. The 2 *A. terricola* Neff genomes that derive from the same isolate are not considered identical in the phylogenomic tree (Fig. 2a). This is not unexpected as the reference genome of this isolate (RefSeq: GCF_000313135.1) has a higher representation of orthologous protein sequences (193 out of 228; supplementary table S3, Supplementary Material online), as compared to the *A. terricola* Neff genome sequenced in this study (188 out of 228; Table 3) in the manually curated database for tree construction (Materials and Methods). Nonetheless, since the genome sequenced in this study is retained in a lower number of scaffolds, future manual curation of the genome annotation will improve its ortholog representation for tree construction. The other amoeba genomes from this study have a much higher representation of orthologs in the tree construction database (between 198 and 224 out of 228; Table 3), comparable to other genomes in the database (supplementary table S3, Supplementary Material online), which, together with the high bootstrap values suggests strong support for their position in the phylogenomic tree (Fig. 2).

### Distinct Codon Usage Patterns among the 6 Amoebae

Before the generation of the high-quality genomes in this study, we were only able to perform codon usage analysis of *A. terricola* Neff (RefSeq: GCF_000313135.1), *Dictyostelium discoideum* AX4 (RefSeq: GCF_000004695.1), and 3 *Naegleria* species (RefSeq: GCF_000004985.1; GCF_008403515.1; GCF_003324165.1) that are not *N. clarki* (supplementary fig. S4, Supplementary Material online). Although *Dictyostelium* is a potential (Abrahão et al. 2018) and *Naegleria* a recently discovered novel giant virus host (Arthofer et al. 2024), we are unquestionably lacking high-quality genomes of other amoebae as currently most publicly available genomes in are simply too fragmented to generate reliable genome assemblies and annotations.

**Fig. 3.**
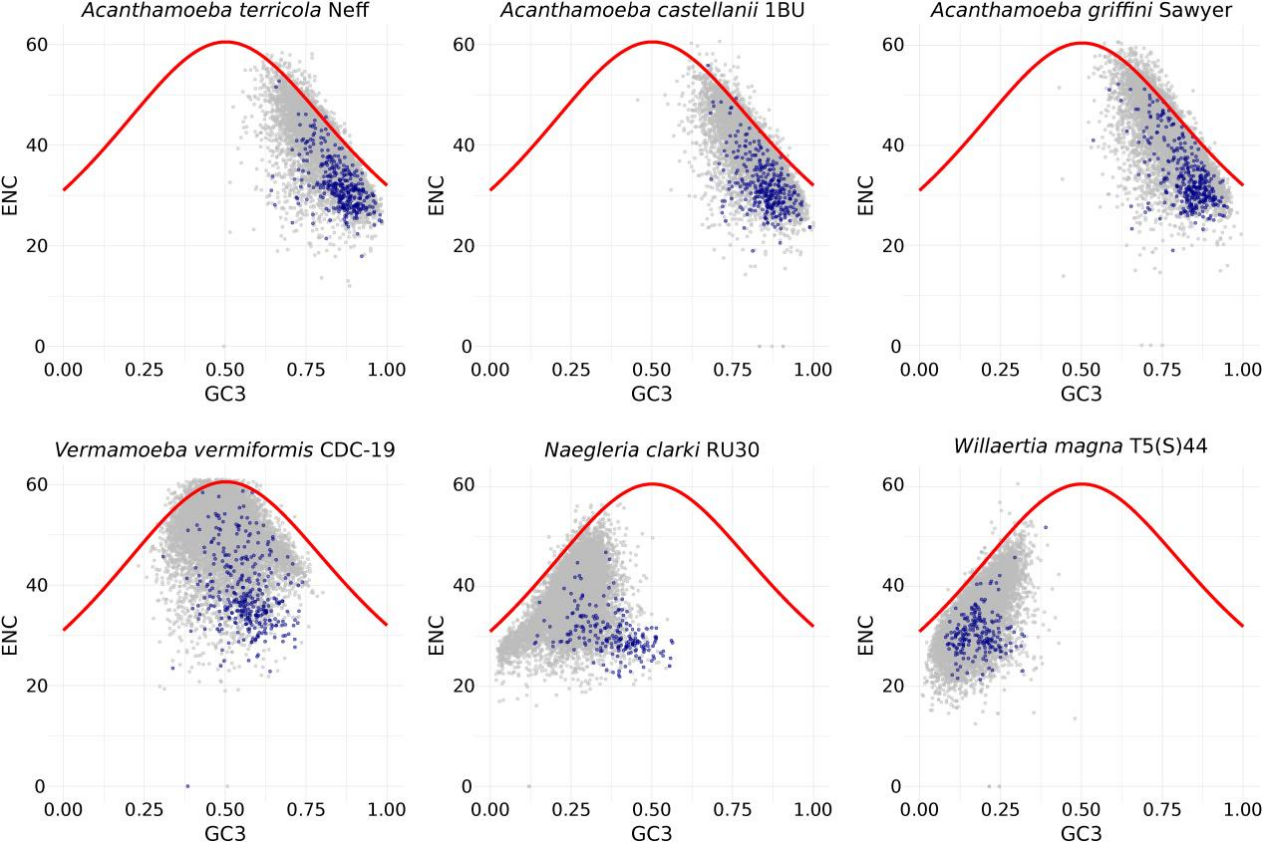
Distinct codon usage patterns across 6 amoebae genomes. ENC values were plotted against GC content at the third codon position (GC3). Each gray or blue dot represents a gene (*A. terricola*: *N* = 11,217, *A. castellanii*: *N* = 11,903, *A. griffini*: *N* = 13,504, *V. vermiformis*: *N* = 14,170, *N. clarki*: *N* = 21,306, *W. magna*: *N* = 18,192). The blue dots represent the top 5% of fragments per kilobase million values from the corresponding transcriptome assemblies as a proxy for identifying highly expressed genes. The continuous red curves represent the relationships between ENC and GC3 under the null hypothesis of no translational selection. If a particular gene lies on the red curve, it is suggested that it is subjected to mutational bias only (i.e. G+C compositional constraints). These plots show that the 6 protists have distinct codon usage patterns at the genus level (i.e. *Acanthamoeba*, *Vermamoeba*, *Naegleria*, and *Willaertia*).

Using the additional 5 amoeba genomes generated here, we applied different methods to compare their codon usage. First, we analyzed the relationship between the effective number of codons used in a gene (ENC) and G+C content in the third codon position (GC3). We used these values to construct ENC plots, allowing for intraspecific and interspecific comparisons of codon usage patterns (Wright 1990). The ENC plots in Fig. 3 show that the 6 amoebae have distinct codon usage patterns at the genus level. The 3 *Acanthamoeba* strains have a similar GC3 composition, with most codons being G- or C-ending. Opposite to *Acanthamoeba*, *N. clarki*, and *W. magna* have low GC3 values. *Vermamoeba vermiformis* has a considerable variation in GC3 values, ranging from 0.23 to 80, most likely reflecting variation in mutational bias among different regions of the genome. Except for *V. vermiformis*, the other 5 amoebae have a clear GC3 preference, while all 6 amoebae have a wide range of ENC values. Genes with low ENC values are often highly expressed (Wright 1990; Mohasses et al. 2020) (blue data points in Fig. 3), as these genes usually use the minimal subset of codons that are recognized by the most abundant tRNA species (Puigbò et al. 2007). These results suggest that for the 6 amoebae, besides mutation, there is translational selection acting for the usage of preferred codons by highly expressed genes.

### Distinct Codon Usage Preferences between Giant Viruses and Their Known Hosts

**Fig. 4.**
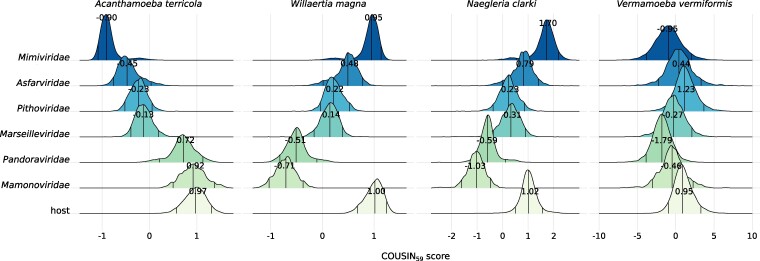
Density curves of the COUSIN_59_ score for giant viruses relative to their known and possible hosts. The density curves are organized by viral family (*Mimiviridae*: *N_viruses_* = 29, *N_CDS_* = 28969, *Asfarviridae*: *N_viruses_* = 9, *N_CDS_* = 4292, *Pithoviridae*: *N_viruses_* = 6, *N_CDS_* = 3393, *Marseilleviridae*: *N_viruses_* = 10, *N_CDS_* = 5735, *Pandoraviridae*: *N_viruses_* = 8, *N_CDS_* = 8532, *Mamonoviridae N_viruses_* = 2, and *N_CDS_* = 890). The bottom density curve always indicates the scores for the host, the name of which is indicated on top of each plot. The 3 lines within the density curves indicate the 95% confidence interval. The numbers within each curve indicate the center values estimated by the Huber M-estimator of location. The COUSIN scores can be interpreted as follows: a score of 1 indicates that the codon usage preferences of viruses are similar to those of the corresponding host; a score of 0 indicates that there is equal usage of synonymous codons; above 1 indicates that codon usage preferences are similar but of larger magnitude (meaning that the codons that are most frequently used in the host are used even more frequently in the virus); between 0 and 1 indicates codon usage preferences are similar but of smaller magnitude (meaning that the codons that are less frequently used in the host are used even less frequently in the virus); below 0 means that the codon usage preferences of viruses are opposite to those of the corresponding host. Since the results for all 3 *Acanthamoeba* species were similar, only the results for *A. terricola* Neff are shown here. Note that the COUSIN_59_ score for *N. clarki* and *V. vermiformis* is depicted on a different scale for visibility.

We then investigated whether codon usage preferences could be used to computationally predict the host range of giant viruses. To be able to compare the codon usage preferences for viruses to those of the 6 amoebae, we calculated the codon adaptation index (CAI) (Sharp and Li 1987; Puigbò et al. 2008) and COdon Usage Similarity Index (COUSIN) scores (Bourret et al. 2019). Using these 2 indices, we compared all available full-length known and possible amoebae-infecting *Nucleocytoviricota* (supplementary tables S4, Supplementary Material online) at both the family (Fig. 4) and genus (supplementary figs. S5 to S10, Supplementary Material online) levels to each host. We generally observed a high correlation between the CAI and COUSIN scores (supplementary table S5, Supplementary Material online). Since the COUSIN scores allow for comparison between organisms (Bourret et al. 2019), we only show the COUSIN_59_ scores in the main figures.

The most intriguing result of this analysis is that viruses belonging to the *Mimiviridae* family (Huber M-estimator of location: −0.898, median absolute deviation or MAD: 0.123) have opposite codon usage preferences to their best-known host *A. terricola* Neff (Huber-M: 0.968, MAD: 0.224; location difference = 1.862, *P* < 2.2e-16; supplementary table S6, Supplementary Material online; Fig. 4), whereas *Mimiviridae* (Huber-M: 0.947, MAD: 0.126) have close (but still significantly different) codon usage preferences to *W. magna* (Huber-M: 1.001, MAD: 0.170; location difference = 0.064, *P* < 2.2e-16; supplementary table S6, Supplementary Material online; Fig. 4), a potential (Abrahão et al. 2018) but yet unknown giant virus host. When we follow the theory that there is translational selection for adaptation of viral codon usage to those of their hosts, our results suggest that Mimiviruses have low fitness in *Acanthamoeba* hosts and high fitness in *W. magna*. However, Mimiviruses have been mainly isolated with *Acanthamoeba* hosts, and attempts to isolate giant viruses with *W. magna* have been unsuccessful so far (Boudjemaa et al. 2020). As reported previously, the *W. magna* genome contains genes related to viral sequences, with the majority being sequences from members of the *Mimivirdae* family (Hasni et al. 2019). These points toward horizontal gene transfer (HGT) events and past infections of mimiviruses in *W. magna*. However, the only mimivirus known to establish a productive infection in *W. magna* is *Tupanvirus soda lake* (Abrahão et al. 2018). Nevertheless, the viral titer increase over a 24-h time period is 6 times lower in this host than in *A. terricola*. *Tupanvirus soda lake* has the largest translational apparatus (including a full set of tRNAs) within the known virosphere (Abrahão et al. 2018), suggesting that Tupanvirus presumably depends less on the host translation system as compared to other viruses. Therefore, it is unsurprising that Tupanvirus can thrive well in *A. terricola*. However, for Tupanvirus and other mimiviruses (with less encoded tRNAs), it remains to be investigated how exactly the translation-related genes are involved in coping with codon usage differences with their host(s).

Another intriguing result is that members of the *Mimiviridae* family (Huber-M: 1.697, MAD: 0.294) have codon usage preferences that are similar but of larger magnitude (i.e. codons that are most frequently used in the host are used even more frequently in the virus) to *N. clarki* (Huber-M: 1.015, MAD: 0.264; location difference = −0.684, *P* < 2.2e-16; supplementary table S6, Supplementary Material online; Fig. 4, supplementary fig. S9, Supplementary Material online) and other *Naegleria* host species (supplementary fig. S4, Supplementary Material online). This indicates that mimiviruses are super-optimized to *Naegleria,* where theory suggests efficient viral replication and high gene expression levels in this host. A recent study showed that a novel giant virus isolate (*Catovirus naegleriensis*, family: *Mimiviridae*), isolated with *N. clarki* as a bait, is specific to *Naegleria* host species, and does not induce infection phenotypes in the “typical” Mimivirus hosts *A. terricola* and *V. vermiformis* (Arthofer et al. 2024). Interestingly, *Catovirus naegleriensis* is only able to infect *Naegleria* host species under xenic conditions (i.e. with bacteria as a food source) but not under commonly used axenic conditions (Arthofer et al. 2024). For all future giant virus isolation studies, more natural culture conditions should be considered, as they can drastically change the infection outcome and have the great potential to reveal novel and natural giant virus hosts.

### Integrated Viral Sequences in the Host Genomes Reveal Potential Novel Virus–Host Associations

It has been recently shown that large parts of protist genomes are of viral origin and that most of these viral integrations appear to be functional viruses (Bellas et al. 2023). These endogenous viral elements (EVEs) comprise virophages, Polinton-like viruses (PLVs), and related entities (Bellas and Sommaruga 2021; Bellas et al. 2023) and are comparable to prophage integrations in bacterial genomes. Although EVEs in eukaryotic genomes were previously thought to be self-synthesizing transposons (Kapitonov and Jurka 2006), the detection of virus hallmark genes (e.g. capsid proteins and packaging ATPases) now suggest that many of these are endogenous viruses (Krupovic et al. 2014; Barreat and Katzourakis 2021; Starrett et al. 2021).

In a previous study, endogenous virus MCP sequences were found in the assemblies of different *Acanthamoeba* species (*A. healyi*, *A. lenticulata*, *A. lugdunensis*, *A. mauritaniensis*, *A. pearcei*, *A. polyphaga*, *A. quina*, *A. rhysodes*, and *A. royreba*), including 2 *A. castellanii* strains (Namur and astronyx), but not in the 2 *A. terricola* Neff strains that were interrogated (WGS accession: AHJI01000000 and AEYA01000000) (Bellas et al. 2023). However, in another study, seven MCP copies were detected in the *A. terricola* Neff reference genome assembly (Maumus and Blanc 2016). The assemblies of *V. vermiformis* isolate TW EDP1, different *Naegleria* species (*N. fowleri*, *N. gruberi*, and *N. lovaniensis*) and *W. magna* were also examined, but no endogenous MCP sequences were found (Bellas et al. 2023). Yet, most of these genomes have been generated with short-read data only, making the detection of EVEs challenging as they are often hidden in repetitive and difficult-to-assemble regions.

The long-read data produced in this study facilitated the detection of MCP sequences in *Acanthamoeba*, as we found seven MCP sequences integrated in *A. terricola* Neff, 3 in *A. castellanii* 1BU, and 5 in *A. griffini* using DIAMOND BLASTX (Buchfink et al. 2015) and profile Hidden Markov Model (HMM) based searches (supplementary tables S7 and S8, Supplementary Material online). We did not detect any integrated MCP sequences in the *V. vermiformis* and *N. clarki* genomes, and only one in the *W. magna* genome, which might well reflect a bias toward the few viruses represented in our database that are known to infect these hosts. Only 1 of the 16 MCPs detected in this study was previously identified. This MCP in *A. terricola* Neff (supplementary table S7 and S8, Supplementary Material online: contig_164) is identical to one of the MCPs detected in Maumus and Blanc (2016) (ACA1_363120), with similarity to the MCP from *Mollivirus sibericum*, isolated from a 30,000-year-old permafrost layer (Legendre et al. 2015). All other (15/16) MCPs detected in this study are novel identifications. Nonetheless, in *A. griffini* (contig_753), we identified a Medusavirus-like MCP that has similarity (supplementary Supplementary Data, Supplementary Material online at Zenodo repository) to some detected previously in *A. mauritaniensis* and *A. lenticulata* (Maumus and Blanc 2016). However, in this foregoing study, the detected MCPs were identified as coming from an undiscovered *Nucleocytoviricota* clade, as medusaviruses were yet to be discovered.

**Fig. 5.**
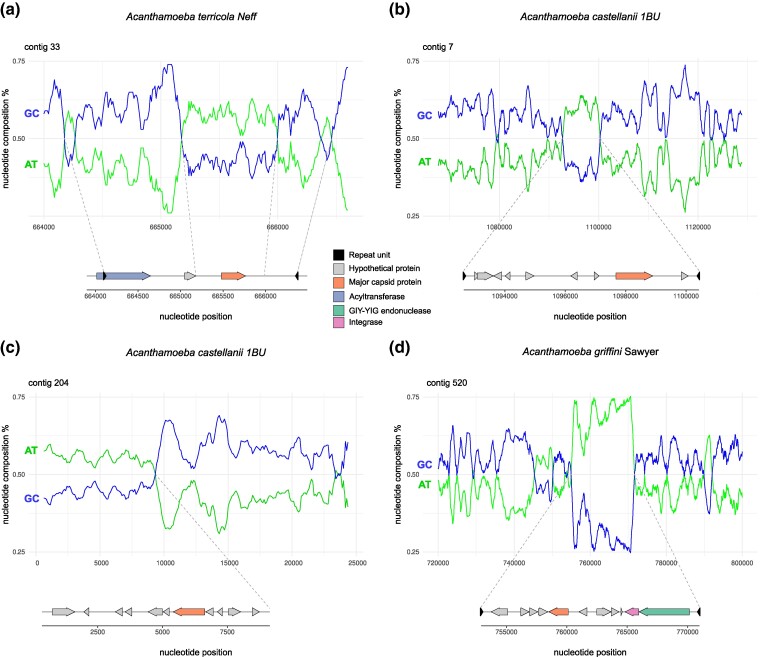
Examples of integrated viral sequences in *acanthamoeba* spp. genomes. In these cases, the integrated viral regions exhibit a notable difference in GC content from the host genome a–d). They are flanked by terminal inverted repeats a, b, and d). The integrated viral regions shown here all contain viral major capsid proteins of unknown origin. The graphs show nucleotide position (bp) in each contig on the *x* axis and GC and AT percentages on the *y* axis. Below each graph, a gene plot and predictions for the integrated viral region are shown. The contig in panel c) is incomplete; therefore, only a part of the integrated viral region was detected, and thus, the detection of terminal inverted repeats was not possible.

Of all detected EVEs (supplementary tables S7 and S8, Supplementary Material online), 4 demonstrated a notable difference in GC content at the site of insertion compared to the genomic GC content (Fig. 5). These differences are only apparent if integrated viruses have different codon usage preferences compared to those of their hosts. The inserted viral regions were flanked by terminal inverted repeats, supporting the hypothesis that these are genuine viral insertions. Interestingly, all 4 EVEs shown in Fig. 5 gave a hit against yet unnamed viral MCPs identified through a previous analysis of all protist assemblies in the Genbank Whole Genome Shotgun database (Bellas et al. 2023). We confirmed these genes as MCPs by modelling their protein structures using AlphaFold. All 4 MCPs gave the best hit against the virus major capsid protein of *Paramecium bursaria Chlorella virus* type 1 (PBCV-1) (Nandhagopal et al. 2002). This virus infects the green algae *Chlorella* that can reside within the protist *Paramecium bursaria*, and has not (yet) been found associated with *Acanthamoeba* spp. The positive AlphaFold hits against PBCV-1 may simply reflect that this is one of the few *Nucleocytoviricota* members for which a high-resolution MCP structural reference is available (Fang et al. 2019; Shao et al. 2022). Other detected EVEs gave reliable BLAST and HMM hits against the MCPs of *A. castellanii medusavirus* and *Mollivirus kamchatka* (supplementary tables S7 and S8, Supplementary Material online). Medusaviruses (*Mamonoviridae*) and molliviruses (*Pandoraviridae*) have close codon usage preferences to *Acanthamoeba* spp. (Fig. 4; supplementary figs. S5 to S7, Supplementary Material online, supplementary table S6, Supplementary Material online), and are known to infect *A. terricola* Neff. However, our analyses suggest that HGT events have also occurred between these viruses and other *Acanthamoeba* spp., suggesting these as possible alternative common hosts.

Not all MCPs detected in this study appear to be intact genes, where stop codons and frameshifts suggest pseudogenization of these. Only 4/7 integrated MCP genes in *A. terricola* Neff, 1/3 in *A. castellanii* 1BU, 4/5 in *A. griffini*, and 1/1 in *W. magna* seem intact. While in most cases, error correction of long-read assemblies with short-read data significantly improves the consensus sequence, it is possible that in endogenous viral regions, this method causes genes to look artificially fragmented (Bellas et al. 2023). We, therefore, checked our nanopore long-read assemblies before error correction with the Illumina short-read data. The observed stop codons and frameshifts in the detected MCP genes were still present, suggesting that these are no artifacts and that some of the integrated MCP genes have been degraded (supplementary table S7, Supplementary Material online). From the examples shown in Fig. 5, the only intact MCP is the one detected in the integrated viral region in *A. griffini* (Fig. 5d). The presence of a retroviral integrase and a GIY-YIG endonuclease in the same region, plus the detection of intact and partial homologous MCP copies in other amoeba genomes (supplementary table S7, Supplementary Material online), suggests that this viral region can still actively move within and between genomes.

## Conclusion

The little information we currently have about the natural host range of giant viruses and the lack of high-quality host genomes is a hurdle for studying virus–host interactions. This study presents 6 amoeba genomes, of which 5 are known giant virus hosts. By comparing codon usage preferences of viruses and hosts, we demonstrate that this measure alone is not a good indicator for predicting giant virus host range. While it has already been reported previously that certain mimiviruses have highly dissimilar codon usage preferences to those of their host *A. castellanii* (Colson et al. 2013), this is the first study that performs a broad-scale analysis including all giant viruses with available full-length genomes, at the time of analyses, and their potential amoebal hosts, for which the high-quality genomes sequenced in this study where required. Indeed, we also find that the currently best-studied giant virus family (*Mimiviridae*) has codon usage preferences opposite those of their best-known laboratory hosts (*Acanthamoeba* spp.). However, this mismatch is not restricted to mimiviruses: our analyses reveal a widespread codon usage mismatch between giant viruses and their hosts. Despite this mismatch, giant viruses can maintain high viral fitness in these hosts. While for most giant viruses, alternative good matching hosts remain to be elucidated, the opposite also seems to occur; a good match in codon usage preferences can also result in low viral fitness (e.g. Tupanvirus in *W. magna*). Therefore, different giant viruses must have adopted different strategies to replicate and maintain high viral fitness in their mismatching hosts.

Notably, the extent of codon usage adaptation of viruses cannot be solely explained by a simple adaptation to the codon usage of their hosts as it reflects a combination of multiple selective and mutational pressures. For example, the host immune system also plays an important role, where immune defenses drive viral codon usage away from sequences detected by the host (Lin et al. 2020). The replication site, nuclear or cytoplasmic, is also an important determinant of viral codon usage. Nuclear viruses (such as medusa-, molli-, and pandoraviruses) tend to have a higher GC content for efficient nuclear export (Mordstein et al. 2020). Indeed, our analyses revealed that the nuclear viruses (*Pandoraviridae* and *Mamonoviridae* in Fig. 4 and Pandoravirus and Mollivirus in supplementary figs. S5 to S7, Supplementary Material online) have closer codon usage preferences to their *Acanthamoeba* hosts (that have a high GC content themselves), as compared to the cytoplasmic viruses. However, most of the giant viruses we know to date replicate in viral factories within the cytoplasm of their host cells (such as mimi-, marseille-, and pithoviruses) and do not have this selective pressure for a higher GC content. Therefore, these viruses may be able to afford such a conflict in codon usage with their hosts.

Interestingly, many viruses—including giant viruses—induce translational shutdown of their hosts (Bercovich-Kinori et al. 2016; Abrahão et al. 2018; Hsu et al. 2021). For phages, it has been shown that this phenomenon influences the selection on viral codon usage, leading to changes in demand for specific tRNAs during the course of infection and driving the acquisition of these tRNAs in viral genomes (Yang et al. 2021). In addition, the presence of translation-related genes may be a good strategy for giant viruses to suboptimize their codon usage preferences and thereby avoid the host immune response. However, the translation-related genes present in viral genomes are not necessarily directly involved in compensating for codon usage differences with their hosts. Another strategy described for phages is to use tRNAs as a viral defense system, where phage-encoded tRNAs counteract tRNA-depleting strategies employed by enzymes from the host to defend from viral infection (van den Berg et al. 2023). The presence of few to many tRNAs in certain giant virus genomes (Koonin and Yutin 2018) could help these viruses escape mutational pressures to adapt their codon usage preferences to that of their hosts.

While predicting virus–host pairs for giant viruses remains challenging, we can get good indications of viable pairs when taking into account additional information. Apart from codon usage preferences, the presence of viral integrations into the host genomes and host integrations into the viral genomes are good indicators of at least past interactions. The amoeba genomes presented in this study set the stage for future experimental studies to understand better how giant viruses interact with their hosts, bringing us a step closer to understanding the natural host range of giant viruses and their determining factors.

## Materials and Methods

### Strains and Growth Conditions

Amoebae were cultured in 75 and 175 cm^2^ culture flasks (Thermo Scientific cat nos. 156472 and 159920). *Acanthamoeba terricola* Neff (ATCC 30010) and *A. castellanii* 1BU (ATCC PRA-105) cells were grown axenically in peptone-yeast extract-glucose medium (PYG: ATCC Medium 712) at 25 °C. *Acanthamoeba griffini* Sawyer (ATCC 50702) cells were grown axenically in PYG medium at 28 °C. *Vermamoeba vermiformis* CDC-19 (ATCC 50237) cells were grown axenically in serum casein glucose yeast extract medium (SCGYEM: ATCC medium 1021) at 25 °C. *Willaertia magna* T5(S)44 (ATCC 50036) cells were grown axenically in PYG medium plus 10% Fetal Bovine Serum (Gibco cat. no. 26140079) at 30 °C. *N. clarki* RU30 (CCAP 1518/14) cells were grown monoxenically with *Escherichia coli* (strain JW5503-1 *ΔtolC732::kan*) in Page's Amoeba Saline buffer (PAS: ATCC medium 1323) at 25 °C. *Escherichia coli* was cultured in lysogeny broth medium (10 g/L NaCl, 10 g/L tryptone, 5 g/L yeast extract) at 37 °C overnight with shaking at 200 rpm. *Escherichia coli* was stored at 4 °C upon usage.

### Nucleic Acid Extractions and Genome Sequencing

For DNA extraction, the amoeba cells were collected by centrifugation for 5 min at 10,000 × *g*. The cells were washed with PAS. High-molecular-weight (HMW) DNA were extracted using the Wizard® HMW DNA Extraction Kit (Promega cat. no. A2920) according to the protocol for plant tissue with the following specifications/modifications: in step 3, the incubation at 65 °C was done for 25 min; in step 4, 5 µL of RNAse A was added, after mixing incubation at 37 °C was done for 25 min; in step 5, 40 µL of Proteinase K Solution was added, after mixing incubation at 56 °C was done for 25 min. For each amoeba, 3 to 5 individual HMW DNA extractions were combined. The DNA was further purified using Agencourt® AMPure® XP beads (Beckman Coulter cat. no. A63882) for subsequent long-read sequencing with the Oxford Nanopore Sequencing Technology. A test run was first done with 4 samples using the MinION platform, and the final sequencing of all 6 samples was done using the PromethION platform. Base calling was done using Guppy v.5.0.7 and the super-accuracy model. DNA extracted using the same method was also used for short-read Illumina sequencing using the NovaSeq SP PE250 readmode.

For RNA extraction, the amoeba cells were collected by centrifugation for 5 min at 10,000 × *g* at 4°C. The cells were washed with cold PAS and after another centrifugation step the cell pellets were resuspended in 1 mL TRIzol Reagent (Invitrogen catalog no. 15596026). The cells were lysed by transferring the samples to Lysing Matrix E tubes (MP Biomedical cat. no. 116914500) and vortexing for 2 min. After transferring the homogenate to a clean tube, the samples were incubated for 5 min at room temperature and then centrifuged for 10 min at 12,000 × *g* at 4 °C to eliminate small beads and cell debris. After transferring the supernatant to a clean tube, 200 µL of phenol/chloroform/isoamyl alcohol (Carl Roth cat. no. A156.2) was added, and the samples were shaken vigorously for 20 s. After incubation at room temperature for 2 to 3 min, the samples were centrifuged for 18 min at 10,000 × *g* at 4 °C. The aqueous phase was transferred to a clean tube, an equal volume of absolute ethanol (Fisher Scientific cat. no. 10644795) was added, and the samples were mixed. The samples were loaded into columns from the RNeasy Mini Kit (Qiagen cat. no. 74104) for subsequent total RNA extraction following the standard protocol. Poly(A) mRNA short-read Illumina sequencing was performed using the NovaSeq SP PE150 readmode.

### Genome Assembly

The long nanopore reads were basecalled using Guppy v5.0.7 (Wick et al. 2019) with the super-accuracy model, and adapter trimmed with Porechop v0.2.4 (Wick et al. 2017). The short DNA and mRNA Illumina reads were quality trimmed with FASTX-Toolkit v0.0.14 (Hannon 2010) and PRINSEQ-lite v0.20.4 (Schmieder and Edwards 2011). Initial long-read assemblies were done using Flye v2.9 (Kolmogorov et al. 2019), comparing the –nano-raw and –nano-hq options. These initial assemblies were compared to hybrid short and long-read assemblies using MaSuRCA v4.0.6. (Zimin et al. 2013). The overall best results were obtained using Flye v2.9 with the –nano-raw option, and these assemblies were used for downstream processing. The assemblies were manually curated by using blast-based searches against sequences of closely related organisms (Table 2), creating lists of known and unknown contigs. From the unknown lists, contaminants, contigs with a low coverage (<*Q*_1_) and short contigs (<1,000 bp) were removed. The long nanopore DNA reads were mapped against the curated assemblies using Minimap2 v2.24 (Li 2018, 2021) and the short Illumina DNA reads were mapped using Bowtie2 v2.5.0 (Langmead and Salzberg 2012). Processing of the alignment files was done using SAMtools (Danecek et al. 2021). To remove spillover contaminants from the sequencing run, for each amoeba the mapping was done against the corresponding nuclear genome concatenated with all 6 mitochondrial genomes. The reads that mapped concordantly were extracted for separate long-read re-assemblies of the nuclear and mitochondrial genomes using Flye v2.9.1, followed by sequence correction using Medaka v1.7.2 (Medaka, 2017/2023), followed by a polishing step using Polypolish v0.5.0 (Wick and Holt 2022).

**Table 2:** Protein and nucleotide databases used for manual curation and gene prediction on genome assemblies

Species	Organism used as reference	Source and identifier	No. of proteins
*A. terricola* Neff	*A. terricola* Neff	RefSeq: GCF_000313135.1	14,969
*A. castellanii* 1BU	*A. terricola* Neff	RefSeq: GCF_000313135.1	14,969
*A. griffini* Sawyer	*A. terricola* Neff	RefSeq: GCF_000313135.1	14,969
*V. vermiformis* CDC-19	*V. vermiformis*	NCBI Taxonomy ID: 5778	212
*N. clarki* RU30	*Naegleria gruberi*	RefSeq: GCF_000004985.1	15,711
	*Naegleria lovaniensis*	RefSeq: GCF_003324165.1	14,755
	*Naegleria fowleri*	RefSeq: GCF_008403515.1	13,816
*W. magna* T5(S)44	*W. magna*	GenBank: KX506079.1; GenBank: KX506077.1	2
	*Naegleria gruberi*	RefSeq: GCF_000004985.1	15,711
	*Naegleria lovaniensis*	RefSeq: GCF_003324165.1	14,755
	*Naegleria fowleri*	RefSeq: GCF_008403515.1	13,816

### Gene Prediction and Annotation

Repeats were identified and masked using RepeatModeler v2.0.4 (Flynn et al. 2020) and RepeatMaster v.4.14 (Tarailo-Graovac and Chen 2009). The short mRNA Illumina reads were mapped against the polished genome assemblies using HISAT2 v2.2.1 (Kim et al. 2019), and Trinity v2.15.0 (Haas et al. 2013) was used to generate a genome-guided de novo transcriptome assembly. Transcript abundance was estimated using RSEM v1.3.3 (Li and Dewey 2011), best hit isoforms were filtered with Trinity and mapped against the polished genome assemblies using GMAP v2021-12-17 (Wu and Watanabe 2005). Processing of the alignment files was done using SAMtools (Danecek et al. 2021). Gene prediction was done using BRAKER2 (Brůna et al. 2021) using a combination of RNA-seq and protein data. For the RNA-seq data, both the full transcriptome and the filtered isoform alignments were used as input. For the protein data, we used available datasets of the same or closely related species (Table 2). Gene annotation was done using Funannotate v1.8.13 (Palmer and Stajich 2023). The predicted proteins were fed to InterProScan v5.60 (Jones et al. 2014), eggNOG-mapper v2.1.10 (Cantalapiedra et al. 2021), Phobius v1.01 (Käll et al. 2004), and SignalP 6.0 (Teufel et al. 2022) to generate functional annotations. Ribosomal RNA and tRNA genes were annotated separately using Infernal v1.1.3 (Nawrocki and Eddy 2013) and tRNAscan-SE v2.0.12 (Chan et al. 2021), respectively. Mitochondrial genome annotation was done using the MITOS webserver (Bernt et al. 2013), followed by manual curation. Genome completeness scores were estimated with BUSCO v.5.4.7 (Simão et al. 2015), using lineage dataset eukaryota_odb10 (eukaryota, 2020-09-10).

### Phylogenetic and Phylogenomic Analyses

Protist ribosomal RNA (rRNA) sequences with a minimum sequence length of 2,000 bp were collected from the PR^2^ reference sequence database (Guillou et al. 2013). The R package *pr2database* was used to select 18S rRNA sequences from specific groups of taxa (phyla *Discosea*, *Heterolobosea*, and *Tubulinea*), reference sequences of the major taxa within these groups, and sequences that are annotated in EukRibo v2 Berney et al. (2022). For all amoebae sequenced, additional almost complete 18S rRNA sequences were added to cross-validate the specific strains we sequenced. Duplicate sequences were removed, and the remaining sequences were aligned with MAFFT v7.490 (Katoh and Standley 2013) using the E-INS-i algorithm. The rRNA sequences in the nuclear genomes of the sequenced amoebae were detected using *cmsearch* within Infernal v1.1.4 (Nawrocki and Eddy 2013) and the Rfam models RF01960, RF00002, and RF02543 for identification of 18S, 5.8S, and 28S rRNA sequences, respectively. The 18S rRNA sequences were extracted from contigs that contain all rRNAs and added to the corresponding alignments using the *–addfragments* options within MAFFT. The alignments were visually checked and, if necessary, manually curated using AliView (Larsson 2014). The alignments were filtered using Gblocks v0.91b (Castresana 2000) (parameters -t = d, -b1 = (½ × N) + 1, -b2 = (½ × N) + 1, -b3 = 8, -b4 = 3, -b5 = a, -b0 = 3). Phylogenetic trees of the 18S rRNA alignments were constructed using IQ-TREE v2.0.7 (Minh et al. 2020), with ModelFinder (Kalyaanamoorthy et al. 2017) to select the optimal model of sequence evolution and 1,000 nonparametric bootstraps.

Phylogenomic analyses were done using PhyloFisher v1.2.11 (Tice et al. 2021), which includes a manually curated database of 240 protein-coding genes from 304 eukaryotic taxa. The standard PhyloFisher workflow was followed to collect putative homologs from the input taxa (*config.py*, *fisher.py*, *informant.py*, *working_dataset_constructor.py*) and to prepare single-protein trees (*sgt_constructor.py*). The single-protein trees were prepared for manual inspection (*forest.py*) with the standalone version of ParaSorter v1.0.4 from PhyloFisher. Orthologs and paralogs were identified during manual inspection, and orthologs were collected. The preliminary statistics after homolog collection show that the data of the newly added taxa is of good quality, which improved after manual curation of the single-protein trees (Table 3). The individual ortholog fasta files for the *Amoebozoa* and *Discoba* clades were selected (*select_taxa.py*) and processed to construct super-matrices using matrix_constructor.py within PhyloFisher. Phylogenomic trees of 228 concatenated protein gene sequences were constructed using IQ-TREE v2.2.2.7 (Minh et al. 2020), with ModelFinder (Kalyaanamoorthy et al. 2017) to select the optimal model of sequence evolution and 1,000 nonparametric bootstraps.

**Table 3:** Statistics of the newly added taxa to the PhyloFisher database. Data before and after manual curation of orthologs is shown

Species	Higher Taxonomy	Lower Taxonomy	Sequences Collected (before curation/after curation)	Genes out of 228 (before curation/after curation)	SBH^a^	BBH^b^	HMM^c^
*A. terricola* Neff	Amoebozoa	Discosea	205/188	189/188	172	0	33
*A. castellanii* 1BU	Amoebozoa	Discosea	216/198	199/198	179	0	37
*A. griffini* Sawyer	Amoebozoa	Discosea	233/203	205/203	194	2	37
*V. vermiformis* CDC-19	Amoebozoa	Tubulinea	246/218	220/218	241	0	5
*N. clarki* RU30	Discoba	Heterolobosea	276/217	226/217	253	8	15
*W. magna* T5(S)44	Discoba	Heterolobosea	252/224	231/224	237	0	15

^a^SBH: Number of collected sequences where the specific query produced a significant hit from the input proteome and collected sequence was a sister to a sequence already in the database of the same higher taxonomy.

^b^BBH: Number of collected sequences where the specific query produced a significant hit and the collected sequence was not a sister to a sequence already in the database of the same higher taxonomy.

^c^HMM: Number of collected sequences for a taxon selected by the default Hidden Markov Model (HMM) route.

### Analysis of Codon Usage Patterns

Codon usage tables, G+C composition, ENC values, CAI, and COUSIN scores for coding sequences (CDSs) of the 6 amoebae were calculated using COUSIN v1.0 (Bourret et al. 2019). ENC quantifies codon usage in a range from extreme bias (ENC of 20: one synonymous codon is used for each amino acid) to no bias (ENC of 61: equal usage of synonymous codons) (Wright 1990). The variation in GC content at the third codon position (GC3) accounts for much of the within-species synonymous codon usage variation in mammals (Ikemura 1985; Aota and Ikemura 1986), and the between-species variation in bacteria (Muto and Osawa 1987). The relationship between GC3 and ENC values was investigated and compared to the null hypothesis (H_0_) of no translational selection. The null hypothesis was calculated as done in (Wright 1990):


H0:ENC=2+s+(29/(s^2+(1-s)^2))

where *s* denotes GC3 scores.

The CAI score quantifies codon usage similarities between a gene and a reference set, with an index ranging from 0 to 1. If a gene always uses the most frequently used synonymous codons in the reference set, the CAI score would be 1. COUSIN also compares the codon usage preferences between a gene and a reference set but normalizes the output over a null hypothesis of equal usage of synonymous codons with an index that can go below 0 or above 1. The COUSIN_18_ variant of the index considers that each of the 18 families of synonymous codons contributes equally to the global index, whereas the COUSIN_59_ variant considers that each family of synonymous codons contributes proportionally to the frequency of the corresponding amino acid in the query (Bourret et al. 2019).

Giant virus genomes from the phylum *Nucleocytoviricota* (supplementary table S4, Supplementary Material online) were collected from the NCBI nucleotide database (https://www.ncbi.nlm.nih.gov/nucleotide/) in July 2021. The taxonomic classification of the collected sequences was done according to the associated GenBank information or deduced from their placement in phylogenetic trees (Schulz et al. 2017; Koonin and Yutin 2018; Yoshikawa et al. 2019; Aylward et al. 2021) and the International Committee on Taxonomy of Viruses (https://ictv.global/). Most sequences were full-length genomes, except for Catovirus CTV1 and Yasminevirus, which are in 2 contigs (supplementary table S4, Supplementary Material online). Unannotated sequences were annotated using Prokka v1.14.6 (Seemann 2014). The CDSs were extracted and used to calculate the codon usage tables and CAI and COUSIN scores at the species, genus and family levels. The Pearson product-moment correlation scores correlation between the CAI and COUSIN were calculated (supplementary table S5, Supplementary Material online). The COUSIN scores were compared using the Huber M-estimator of location and the corresponding MAD scores (Huber and Ronchetti 2009) and Wilcoxon Rank Sum tests with continuity correction (Bauer 1972; Hollander et al. 2015) (supplementary table S6, Supplementary Material online).

### Detection of Integrated Viral Sequences in the Amoeba Genomes

To detect integrated viral sequences in the amoeba genomes, we downloaded 256 MCP genes from full-length *Nucleocytoviricota* genomes from GenBank (May 2023). We concatenated these with 196 MCP genes from virophages plus known PLVs from a previous study (Bellas and Sommaruga 2021). The total of 452 MCP genes were aligned with MAFFT v7.055b (Katoh and Standley 2013) using the E-INS-i algorithm. HMM profiles were constructed with hmmbuild, and the amoeba genomes were interrogated with hmmsearch from HMMER v3.3.2 (http://hmmer.org/). We separately interrogated the amoeba genomes with HMM profiles from the MCPs of PLVs and virophages generated in Bellas and Sommaruga (2021), as well as with HMM profiles from 5 core genes (A23 packaging ATPase, D5-like helicase-primase, DNA polymerase family B, DNA/RNA helicase, poxvirus late transcription factor VLTF3-like) of *Nucleocytoviricota* generated in Schulz et al. (2020). The amoeba genomes were also interrogated with DIAMOND BLASTX v2.1.8.162 (Buchfink et al. 2015) using the *Nucleocytoviricota* MCP database constructed in this study and the latest MCP/PLVs/EVEs database from Bellas et al. 2023 (settings: –evalue 1e-12 –range-culling -F 15 –max-target-seqs 1, and compared with settings: –range-culling -F 15). The contigs with significant hits (supplementary tables S7 and S8, Supplementary Material online) against any of these databases were extracted using fastx_filter v.1.0 (https://github.com/amanzanom/seqTools), re-annotated with prokka v.1.14.6 (Seemann 2014) under the kingdom viruses, and manually inspected.

### Statistics and Data Visualization

The data was processed and visualized using R v.4.3.0 (R Core Team 2023), with the packages dplyr (Wickham et al. 2023), ggplot2 (Wickham 2016), gggenes (Wilkins 2023), ggridges (Wilke 2024), MASS (Venables and Ripley 2002), and viridis (Garnier et al. 2024). Phylogenetic trees were visualized using iTOL v.6.9 (Letunic and Bork 2024). The sliding window analysis for visualizing integrated viral sequences was done using bedtools v.2.30.0 (https://github.com/arq5x/bedtools2). Final figures and graphs were made with Inkscape v.1.1.2 (https://inkscape.org).

## Supplementary Material

evae271_Supplementary_Data

## Data Availability

The raw reads (Illumina, MinION, PromethION) and the genome assemblies are available at NCBI under the following BioProject accession numbers: *A. terricola* Neff: PRJNA1128888; *A. castellanii* 1BU: PRJNA1128890; *A. griffin*i Sawyer: PRJNA1128892; *V. vermiformis* CDC-19: PRJNA1128894; *N. clarki* RU30: PRJNA1128897; and *W. magna* T5(S)44: PRJNA1128895. The annotated amoeba genomes, including the curated mitochondrial genomes, nucleotide and protein alignments, distance matrices, phylogenetic and phylogenomic trees, and codon usage data of amoebae and giant viruses can be found at Zenodo.
